# Supplementary material for: Prediction of All-Cause Mortality Following Percutaneous Coronary Intervention in Bifurcation Lesions Using Machine Learning Algorithms
Source: J Pers Med. 2022 Jun 17;12(6):990. doi: 10.3390/jpm12060990 (PMC9224705; doi:10.3390/jpm12060990)
Supplement: Supplementary file 1 [file jpm-12-00990-s001.zip › jpm-1747321-supplementary.pdf]

SUPPLEMENTARY MATERIALS

Prediction of All-Cause Mortality Following Percutaneous Coronary Intervention in Bifurcation Lesions Using Machine Learning Algorithms

The RAIN-ML prediction model

Index

Study list collaborators ______________________________ page 02

Extended methods ______________________________ page 03

Supplementary Table 1. Characteristics of patients from the discovery cohort ______________ page 07

Supplementary Table 2. Discovery cohort: training *vs*. internal validation dataset ___________ page 09

Supplementary Table 3. Grid Search: Model and Data Imbalance Correction _______________ page 11

Supplementary Table 4. Tuning of the RAIN-ML model ______________________________ page 12

Supplementary Table 5. Characteristics of discovery cohort *vs*. external validation cohort ____ page 13

Supplementary Table 6. Patient distribution and risk of all-cause mortality _________________page 15

Supplementary Table 7. Predictive performance of the RAIN-ML model __________________ page 16

Supplementary Figure 1. Feature selection ______________________________ page 17

List of other collaborators

Antonio Montefusco MD; Mauro Pennone MD (Division of Cardiology, Department of Medical Sciences, University of Turin, Turin Italy)

Giacomo Boccuzzi MD (Dipartimento di Cardiologia, Ospedale San Giovanni Bosco,Turin Italy)

Giorgio Quadri MD, Ferdinando Varbella MD (Department of Cardiology, Infermi Hospital, Rivoli, Italy-Department of Cardiology, San Luigi Gonzaga Hospital, Orbassano, Turin, Italy)

Grzegorz Smolka Prof, Wojciech Wańha MD, (Department of Cardiology, Medical University of Silesia, Katowice, Poland)

Andrea Rognoni MD, Andrea Mennuni MD (Coronary Care Unit and Catheterization laboratory , A.O.U. Maggiore della Carità, Novara, Italy)

Carlo di Mario Prof (Structural Interventional Cardiology, Careggi University Hospital, Florence, Italy)

Francesco Romeo Prof. (Department of Medicine, Università degli Studi di Roma 'Tor Vergata', Rome, Italy)

Filippo Figini MD (Pederzoli Hospital, Peschiera del Garda, Italy)

Zenon Huczek MD (University Clinical Hospital, Warsaw, Poland)

Davide Capodanno Prof, (Division of Cardiology, Cardio-Thoracic-Vascular Department, Azienda Ospedaliero Universitaria "Policlinico-Vittorio Emanuele," Catania, Italy)

Luca Gaido MD (Division of Cardiology, Ospedale Maria Vittoria, Turin)

Luca Benini (DEI, University of Bologna, 40123 Bologna, Italy)

Extended Methods

Study population

1. Discovery cohort

RAIN (veRy thin stents for patients with left mAIn or bifurcatioN in real life) registry

Participating Study Centers

- Coronary Care Unit and Catheterization laboratory , A.O.U. Maggiore della Carità , Novara , Italy
- Structural Interventional Cardiology, Careggi University Hospital, Florence, Italy
- Division of Cardiology, Cardio-Thoracic-Vascular Department, Azienda Ospedaliero Universitaria "Policlinico-Vittorio Emanuele," Catania, Italy
- Department of Cardiovascular Medicine, Nippon Medical School, 1-1-5, Sendagi, Bunkyo-ku, Tokyo, Japan
- Department of Cardiovascular Sciences, IRCCS Centro Cardiologico Monzino, Milan, Italy; University of Milan, Milan, Italy
- Interventional Cardiology, ASST Fatebenefratelli-Sacco, Milano, Italy
- Dipartimento di Cardiologia, Ospedale San Giovanni Bosco, Italy (FU; GB); University Clinical Hospital, Warsaw, Poland
- Department of Cardiology, Hospital Clinico San Carlos, Madrid, Spain
- San Raffaele Scientific Institute, Milan, Italy (SM, AC); Pederzoli Hospital, Peschiera del Garda, Italy
- Department of Cardiology, Medical University of Silesia, Katowice, Poland
- Department of Cardiology, Infermi Hospital, Rivoli, Italy- Department of Cardiology, San Luigi Gonzaga Hospital, Orbassano, Turin, Italy
- Division of Cardiology, S. Giovanni Evangelista Hospital, Tivoli, Rome, Italy
- Pierre and Marie Curie University, Paris, France (GH), Division of Cardioloy, Universityspirtal of Zurich
- Cardiology Unit, ASST Bergamo Est, Seriate Hospital, Seriate (BG), Italy / Clinical and Interventional Cardiology Unit, Sant'Ambrogio Cardio-Thoracic Center, Milan, Italy
- Cardiology Unit, ASST Fatebenefratelli/Sacco, Sacco Hospital, Milan, Italy
- Cardiology Unit, ASST Milanese Ovest, Magenta Hospital, Magenta (MI), Italy
- Cardiology Unit, ASST Milanese Ovest, Legnano Civil Hospital, Legnano (MI), Italy
- Cardiology Unit, ASST Fatebenefratelli/Sacco, Fatebenefratelli Hospital, Milan, Italy

Inclusion criteria

The study population include pts aged over 18 y.o. enrolled with informed consent, based on the following inclusion criteria: clinical indication to PCI, Complex coronary lesions, borne by unprotected left main or bifurcation, execution of PTCA, followed by the implantation of a latest generation ultra-thin stent:

a) Platinum-chromium coated with a permanent polymer loading everolimus with strut thickness of 81 μm for diameters from 2.25 to 3.5 mm (Promus Element®, Boston Scientific
b) Cobalt-chromium coated with a permanent polymer loading everolimus with a strut thickness of 80 μm (Xience Alpine®, Abbot);
c) Cobalt-chromium coated with a biodegradable polymer loading sirolimus with strut thickness of 80 μm; (Ultimaster®, Terumo Corporation);
d) Platinum-chromium coated with a biodegradable polymer loading everolimus with strut thickness of 74 μm for diameters in the range 2.25-2.75 mm, 79 μm for diameters in the range 3.00-3.50 mm, and 81 μm for diameter equal to 4.0 mm; (Synergy®, Boston Scientific);
e) Platinum-chromium coated with a biodegradable polymer loading zotarolimus with a strut thickness of 74 μm for diameters ≤2·5 mm, (2) 79 μm for diameters in the range 3.0-3.50 mm, and (3) 81 μm for diameter equal to 4.0 mm (Resolute Onyx®, Medtronic).

No exclusion criteria have been considered.

2. External validation cohorts

a. *BIO-RESORT trial cohort*

Participating study Centers

- Thoraxcentrum Twente, Medisch Spectrum, Twente, Enschede
- Twente, Enschede; Rijnstate Hospital, Arnhem
- Haga HospitalThe Hague
- Albert Schweitzer Hospital, Dordrecht

BIO-RESORT study characteristics
Investigator-initiated three-arms trial that assessed two independent non-inferiority hypotheses in allcomers that the 1year safety and efficacy of the biodegradable polymer everolimus-eluting stent is non-inferior to the durable polymer zotarolimus-eluting stent, and that the 1-year safety and efficacy of the biodegradable polymer sirolimus-eluting stent is noninferior to the durable polymer zotarolimus-eluting stent.

Inclusion criteria
All-comer patients were eligible if they were aged 18 years or older, capable of providing informed consent, and required a percutaneous coronary intervention with drug-eluting stent implantation according to clinical guidelines or the operators' judgment. All coronary syndromes, de-novo and re-stenotic lesions, and coronary artery or bypass lesions were permitted. There was no limit for lesion length, reference size, number of lesions, or diseased vessels to be treated. The exclusion criteria were: participation in another randomized drug or device study before reaching the primary endpoint of that study; planned surgery necessitating interruption of dual antiplatelet therapy within the first 6 months; known intolerance to components of the investigational product or medication required (eg, intolerance to concomitant anticoagulation or antiplatelet therapy); uncertainty about the adherence to follow-up procedures or an assumed life expectancy of less than a year; or known pregnancy. The trial complied with the CONSORT 2010 Statement and Declaration of Helsinki and was approved by the Medical Ethics Committee Twente and the institutional review boards of all participating centres. All patients provided written informed consent.

b. *DUTCH PEERS (DUrable polymer-based sTent CHallenge of Promus ElemEnt versus ReSolute integrity: TWENTE II) trial cohort*

Participating study Centers

- Thoraxcentrum Twente, Medisch Spectrum, Twente, Enschede
- Twente, Enschede; Rijnstate Hospital, Arnhem
- Haga HospitalThe Hague
- Albert Schweitzer Hospital, Dordrecht

DUTCH PEERS study characteristics
Multicenter, patient-blinded, investigator-initiated, randomized clinical trial of patients with an indication for PCI with DES randomized in a 1:1 fashion for treatment with Resolute Integrity ZES or PROMUS Element EES.

Inclusion criteria
Patients 18 years of age and older and capable of providing informed consent with an indication for PCI with DES. Exclusion criteria were limited and all coronary syndromes, de novo and re-stenotic lesions, and coronary artery or bypass stenosis were permitted. There was no limit for lesion length, reference size, or number of lesions to be treated. Generally, dual antiplatelet therapy consisted of aspirin and clopidogrel and was prescribed in patients without anticoagulation therapy for 1 year. In patients on oral anticoagulation, triple therapy was generally prescribed for 1 to 3 months, followed by a period with clopidogrel as a single antiplatelet agent. Concerning external validation, the variable ejection fraction (EF) in the DUTCH-PEERS and the BIO-RESORT cohorts was reported dichotomously and was thus entered into the model for each patient as a value of either "30%" or "50%" (*see Table 1 and Table S5).

Development of the machine learning model

Machine learning (ML) models were developed in the discovery cohort (n=2,393; randomized according to a ratio of 3:1 in training and internal validation datasets; Table S2) and then tested in the external validation cohort (n=1,701). In particular, each model was built in the training cohort (n=1,795) and then tested in both internal (n=598) and external validation cohorts. An overview of model development is provided in Figure 1A. The structured dataset included 38 features easily available at the end of the bifurcation PCI procedure (15 patient-related and 23 lesion-related parameters; Table S1). Fisher score was used to assess the association of variables to the primary endpoint (2-years all-cause mortality) in the training cohort. Variables with a coefficient >0.75 were selected and introduced in diagnostic modelling analysis (13 patient-related and 12 lesion-related parameters; Figure S1).

Algorithms of supervised ML were applied to formulate predictions on the primary endpoint on the base of a pre-defined set of labeled multi-dimensional paired input-output data. We assessed the diagnostic performance of 5 different ML models, including linear discriminant analysis (LDA), random forest regressor (RF) algorithm, support vector machine (SVM) with different kernels (linear and gaussian radial basis function), and isolation forest classifier [1]. These models display some advantages as compared to a deep neural network (DNN) approach. DNNs have shown excellent performance on homogenous datasets (e.g., image classification, sound generation, natural language processing, medical image analysis, seizure detection); on the contrary their application to tabular data is questioned: (i) Tabular data include small data sets with missing data (state-of-the-art image-classification algorithms are trained on 300 millions of images); (ii) DNNs search for spatial/temporal correlation, while usually there is no spatial correlation between tabular data (e.g., age and gender may not be correlated in any way). (iii) DNNs do not handle categorical data [2]. On the other side, machine leaning algorithms here applied are simpler and may easily handle tabular data. In addition, they may only discriminate patients according to linear and not-linear representations, preventing the creation of complex functions, tailored on training data, and then biased by overfitting effect. LDA employs a linear combination of variables to maximize the separation between groups (Death *vs.* No events), increasing precision estimates by variance reduction. The predicted endpoint is derived from the following equation: Endpoint (all-cause mortality) = $LDAcoeff_1*Variable_1 + LDAcoeff_2*Variable_2 + \ldots + LDAcoeff_n*Variable_n$ > tested thresholds. The RF algorithm creates a pre-defined set of classification trees ("n" classification trees) with a fixed maximum number of splits for each tree. The predicted endpoint results from the outcome of each classification tree of the forest; if at least (n/2)+1 of "n" trees of the RF predicts death as outcome, then this endpoint is assigned to the patient. Linear SVM builds a classification model to assign patients to their outcome given a linear boundary. The model defines the plane which best separates groups of patients (Death *vs.* No events), maximizing the distances between them. Patients are classified according to the following equation: $SVMcoeff_0 + SVMcoeff_1*Variable1 + SVMcoeff_2*variable_2 + \ldots. + SVMcoeff_n*Variable_n$. Gaussian SVM allows to divide patients using a non-linear boundary; the corresponding equation is: $SVMcoeff_0 + SVMcoeff_1*f(Variable1) + SVMcoeff_2*f(variable_2) + \ldots. + SVMcoeff_n*f(Variable_n)$, where "f" is an exponential function coefficient. Isolation forest is a particular type of RF, which uses unsupervised learning to discriminate anomalies (in this case, patients with death occurrence) from normal data (patients without events).

To correct for dataset imbalance (136 death events in the discovery cohort; 5.7%), three different oversampling algorithms were applied to all the models: synthetic minority over-sampling technique (SMOTE), SMOTE and nearest neighbors, and random oversampling. Briefly, oversampling algorithms imputes new simulated patient data starting from real patients from the discovery cohort, in the virtual space created by patient parameters, in order to balance the number of patients with death occurrence and patients

without events at model training. These algorithms avoid the accuracy paradox (a falsely high accuracy due to over-prediction of the most represented class). For further details please refer to Prati *et al.* [3].

A grid search was performed in the training cohort to assess ML models accuracy with or without correction for dataset imbalance (Table S3). RF with random oversampling was the best-performing model and was selected for tuning. Tuning was performed using the patients of the training cohort (n=1,795) by 10-fold cross-validation. The two hyperparameters to be tuned in the RF model were the maximum number of splits (leaves; from 10 to 320) and the number of classification trees in the forest (from 10 to 800). The best RF classification algorithm was composed by 100 classification trees with a maximum number of 10 splits (Table S4).

The 10-fold cross-validation algorithm randomly divides the cohort into 10 groups; then the model is trained within the first 9 groups and validated in the remaining one. The process is reiterated 10 times, with the validation group rotating at each round. Accuracy at validation is calculated from the mean of accuracy obtained at each round [1].

The RF model was then applied to training, internal validation, external validation and mixed (discovery plus external validation) cohorts. The model was developed to predict 2-years all-cause mortality; its performance was then assessed also at different time points (30-day, 1-year). Missing values for the external validation cohort were replaced with mean and standard deviation, (or median and interquartile range, when appropriated), for each single parameter. The discovery cohort did not include patients with missing data.
Overfitting bias was defined as difference between accuracy obtained at training and accuracy at internal or external validation. Overfitting bias was minimized by: (i) Application of machine learning approach which intrinsically reduce overfitting (LDA, RF, SVM); (ii) Correction for dataset imbalance; (iii) Selection of the best model basing on internal validation by 10-fold cross validation without considering accuracy at training.
To confirm the generalizability of the model, we used a k-center cross-validation approach: the model is trained in a cohort composed by patients of all centers except one, and then validated in the remaining center. The process is re-iterated "n" times, where "n" is the number of centers, with the center use for validation rotating at each round. Accuracy at validation is calculated from the mean of accuracy obtained at each round.

Finally, continual learning was applied in order to demonstrate the improvement of ML models over time [4]. Indeed, ML models have the opportunity to improve their classification algorithm by learning strategies, at the increase of enrollment time and number of recruited patients. Learning simulation for the RF model was assessed at the increase of the time of enrollment from 3 to 33 months; 70% of the discovery cohort was used for training (patients enrolled first) and 30% for validation (last enrolled patients). Accuracy, sensitivity, and specificity are reported in Figure 4.

A user-friendly online interface was designed to facilitate the application of the RAIN-ML prediction model in clinical practice (available at https://rain.hpc4ai.it).

REFERENCES

1. Bishop CM. Pattern recognition and machine learning. Springer, 2006.
2. Borisov V. Deep neural networks and tabular data: A survey. arXiv preprint arXiv:2110.01889, 2021.
3. Prati BRC, Monard MC. A study of the behavior of several methods for balancing machine learning training data. SIGKDD Explor. Newsl. 2004. 6:20–29.
4. Ring MB. CHILD: A first step towards continual learning. Learning to learn. Springer, Boston, MA, 1998. 261-292.

Table S1. Characteristics of patients from the discovery cohort

	Variable	Discovery cohort (n=2,393)	Death (n=137)	No events (n=2,256)	*P*-value
Patient parameters	Age (years)	69 [61; 77]	77 [68; 83]	69 [61; 77]	**<0.001**
	Sex (ref. male; n, %)	1,819 (76.0)	96 (70.1)	1,723 (76.4)	0.094
	Hypertension (ref. yes; n, %)	1,791 (74.8)	120 (87.6)	1,671 (74.1)	**<0.001**
	Hyperlipidemia (ref. yes; n, %)	1,451 (60.6)	87 (63.5)	1,364 (60.5)	0.479
	Diabetes (ref. yes; n, %)	805 (33.6)	82 (59.9)	723 (32.0)	**<0.001**
	Smoker				0.359
	No (n, %)	1,163 (48.6)	74 (54.1)	1,089 (48.2)	
	Previous (n, %)	729 (30.5)	35 (25.5)	694 (30.8)	
	Current (n, %)	501 (20.9)	28 (20.4)	473 (21.0)	
	CKD (ref. GFR<60 mL/min; n, %)	538 (22.5)	87 (63.5)	451 (20.0)	**<0.001**
	Previous PCI (ref. yes; n, %)	792 (33.1)	38 (27.7)	754 (33.4)	0.170
	Previous CABG (ref. yes; n, %)	126 (5.3)	12 (8.8)	114 (5.1)	0.059
	Previous MI (ref. yes; n, %)	737 (30.8)	57 (41.6)	680 (30.1)	**0.005**
	EF at echo (%)	55 [55; 60]	55 [50; 60]	55 [55; 60]	**0.006**
	PCI indication				**<0.001**
	STEMI (n, %)	419 (17.5)	39 (28.5)	380 (16.8)	
	NSTEMI (n, %)	580 (24.3)	46 (33.6)	534 (23.7)	
	Unstable angina (n, %)	347 (14.5)	13 (9.5)	334 (14.8)	
	Stable angina (n, %)	587 (24.5)	25 (18.2)	562 (24.9)	
	Positive stress test (n, %)	309 (12.9)	11 (8.0)	298 (13.2)	
	Planned angiography (n, %)	151 (6.3)	3 (2.2)	148 (6.6)	
	ACS at presentation (ref. yes; n, %)	1,344 (56.2)	98 (71.5)	1,246 (55.2)	**<0.001**
	STEMI at presentation (ref. yes; n, %)	419 (17.5)	39 (28.5)	380 (16.8)	**0.001**
	Kind of DAT (aspirin plus …)				**0.003**
	Clopidogrel (n, %)	1,561 (65.2)	107 (78.1)	1,454 (64.4)	
	Ticagrelor (n, %)	641 (26.8)	26 (19.0)	615 (27.3)	
	Prasugrel (n, %)	191 (8.0)	4 (2.9)	187 (8.3)	
Lesion parameters	First lesion vessel				**<0.001**
	LM (n, %)	595 (24.9)	62 (45.3)	533 (23.6)	
	LAD (n, %)	1,172 (48.9)	53 (38.7)	1,119 (49.6)	
	Cx/MO (n, %)	418 (17.5)	10 (7.3)	408 (18.1)	
	RCA (n, %)	166 (6.9)	11 (8.0)	155 (6.9)	
	RI (n, %)	42 (1.8)	1 (0.7)	41 (1.8)	
	Lesion localization				**<0.001**
	Ostial (n, %)	85 (3.6)	4 (2.9)	81 (3.6)	
	Proximal (n, %)	732 (30.6)	29 (21.2)	703 (31.2)	
	Middle (n, %)	851 (35.5)	40 (29.2)	811 (35.9)	
	Distal (n, %)	725 (30.3)	64 (46.7)	661 (29.3)	
	Type C lesion (ref. yes; n, %)	897 (37.5)	49 (35.8)	848 (37.6)	0.669
	Severe calcification (ref. yes; n, %)	349 (14.6)	23 (16.8)	326 (14.5)	0.452
	Diffuse disease (ref. yes; n, %)	938 (39.2)	82 (59.9)	856 (37.9)	**<0.001**
	Kind of bifurcation				**<0.001**
	Distal LM (n, %)	655 (27.4)	62 (45.3)	593 (26.3)	
	LAD/Diag (n, %)	1,123 (46.9)	51 (37.2)	1,072 (47.5)	
	Cx/MO (n, %)	450 (18.8)	13 (9.5)	437 (19.4)	
	RCA/PL (n, %)	165 (6.9)	11 (8.0)	154 (6.8)	

	Medina class				
	1,1,1 (n, %)	810 (33.9)	41 (29.9)	769 (43.1)	**0.015**
	1,1,0 (n, %)	785 (32.8)	46 (33.6)	739 (32.8)	
	1,0,1 (n, %)	221 (9.2)	16 (11.7)	205 (9.1)	
	0,1,1 (n, %)	119 (5.0)	7 (5.1)	112 (5.0)	
	1,0,0 (n, %)	199 (8.3)	21 (15.3)	178 (7.9)	
	0,1,0 (n, %)	139 (5.8)	3 (2.2)	136 (6.0)	
	0,0,1 (n, %)	120 (5.0)	3 (2.2)	117 (5.2)	
	Kind of strategy				
	Provisional (n, %)	1,964 (82.1)	113 (82.5)	1,851 (82.0)	0.898
	Two stents (n, %)	429 (17.9)	24 (17.5)	405 (18.0)	
	Use of imaging				
	No (n, %)	1,586 (66.3)	81 (59.1)	1,505 (66.7)	0.188
	IVUS (n, %)	780 (32.6)	54 (39.4)	726 (32.2)	
	OTT (n, %)	27 (1.1)	2 (1.5)	25 (1.1)	
	Predilatation (ref. yes; n, %)	2,090 (87.3)	122 (89.1)	1,968 (87.2)	0.535
	Kind of balloon				
	Conventional (n, %)	2,039 (97.5)	111 (91.0)	1,928 (98.0)	**<0.001**
	Angiosculpt (n, %)	22 (1.1)	6 (4.9)	16 (0.8)	
	Scoring (n, %)	29 (1.4)	5 (4.1)	24 (1.2)	
	Rotablator (ref. yes; n, %)	144 (6.0)	6 (4.4)	138 (6.1)	0.406
	Kind of stent on MB				
	Resolute Onyx (n, %)	692 (29.2)	36 (26.7)	656 (29.3)	0.088
	Xience Alpine (n, %)	590 (24.8)	32 (23.7)	558 (25.0)	
	Synergy (n, %)	505 (21.3)	42 (31.1)	463 (20.7)	
	Ultimaster (n, %)	215 (9.1)	11 (8.1)	204 (9.1)	
	Biomatrix alpha (n, %)	4 (0.2)	0 (0.0)	4 (0.2)	
	Promus (n, %)	365 (15.4)	14 (10.4)	351 (15.7)	
	MB lesion diameter (mm)	3.0 [2.8; 3.5]	3.0 [2.8; 3.5]	3.0 [2.8; 3.5]	0.701
	MB lesion length (mm)	22 [16; 28]	20 [16; 28]	22 [16; 28]	0.347
	MB atmospheres (atm)	14 [12; 16]	14 [12; 18]	12 [12; 16]	**0.019**
	Stent on SB (ref. yes; n, %)	429 (17.9)	24 (17.5)	405 (18.0)	0.898
	SB lesion diameter (mm)	2.5 [1.0; 2.8]	2.5 [1.0; 3.0]	2.4 [1.0; 2.8]	0.372
	SB lesion length (mm)	28 [20; 33]	26 [16; 33]	28 [20; 33]	**<0.001**
	SB atmospheres (atm)	12 [11; 14]	12 [10; 14]	12 [11; 14]	0.453
	POT (ref. yes; n, %)	1,831 (76.5)	104 (75.9)	1,727 (76.6)	0.864
	ATM Post (atm)	20 [16; 22]	18 [16; 22]	20 [16; 22]	0.056
	Final kissing ballon (ref. yes; n, %)	994 (41.5)	56 (40.9)	938 (41.6)	0.871
**Outcome**	Death (ref. yes; n, %)	137 (5.7)	N.A.	N.A.	N.A.
	Mean follow-up at the event (days)	274 [52; 434]			
	Death within 30 days (ref. yes; n, %)	29 (1.2)			
	Death within 1 year (ref. yes; n, %)	89 (3.7)			
	Death within 2 year (ref. yes; n, %)	125 (5.2)			

The table shows patient and lesion parameters in the discovery cohort (n=2,393), and the comparison of patients who died (n=137) compared to those who did not experienced events (n=2,256). Variables are reported as median [interquartile range], or absolute number (percentage, %), as appropriated. Differences were considered significant when $p<0.05$. CKD, Chronic Kidney Disease; PCI, Percutaneous Coronary Intervention; CABG, Coronary Artery Bypass Graft; MI, Myocardial Infarction; EF, Ejection Fraction; STEMI, ST-segment Elevated Myocardial Infarction; NSTEMI, Non ST-segment Elevated Myocardial Infarction; ACS, Acute Coronary Syndrome; DAT, Double Antiaggregant Therapy; LM, Left Main; LAD, Left Anterior Descending; Cx/MO, Circumflex/Marginal; RCA, Right Coronary Artery; RI, Right Intermedius; Diag, Diagonal; PL, Posterior Left; IVUS, IntraVascular UltraSound; OCT, Optical Coherence Tomography; MB, Main Branch; SB, Side Branch; POT, Proximal Optimization Technique.

Table S2. Discovery cohort: training *vs.* internal validation dataset

	Variable	Training dataset (n=1,795)	Internal Validation dataset (n=598)	*P*-value
**Patient parameters**	Age (years)	70 [61; 77]	69 [61; 78]	0.539
	Sex (ref. male; n, %)	1,379 (76.9)	440 (73.6)	0.107
	Hypertension (ref. yes; n, %)	1,331 (74.2)	460 (76.9)	0.176
	Hyperlipidemia (ref. yes; n, %)	1,074 (59.8)	377 (63.0)	0.164
	Diabetes (ref. yes; n, %)	605 (33.7)	200 (33.4)	0.907
	Smoker			0.991
	No (n, %)	872 (48.6)	291 (48.6)	
	Previous (n, %)	548 (30.5)	181 (30.3)	
	Current (n, %)	375 (20.9)	126 (21.1)	
	CKD (ref. GFR<60 mL/min; n, %)	391 (21.8)	147 (24.6)	0.156
	Previous PCI (ref. yes; n, %)	605 (33.7)	187 (31.3)	0.273
	Previous CABG (ref. yes; n, %)	97 (5.4)	29 (4.8)	0.599
	Previous MI (ref. yes; n, %)	554 (30.9)	183 (30.6)	0.905
	EF at echo (%)	55 [55; 60]	55 [55; 65]	0.951
	PCI indication			0.530
	STEMI (n, %)	305 (17.0)	114 (19.1)	
	NSTEMI (n, %)	447 (24.9)	133 (22.2)	
	Unstable angina (n, %)	257 (14.3)	90 (15.1)	
	Stable angina (n, %)	440 (24.6)	147 (24.5)	
	Positive stress test (n, %)	227 (12.6)	82 (13.7)	
	Planned angiography (n, %)	119 (6.6)	32 (5.4)	
	ACS at presentation (ref. yes; n, %)	1,007 (56.1)	337 (56.4)	0.914
	STEMI at presentation (ref. yes; n, %)	305 (17.0)	114 (91.1)	0.248
	Kind of DAT (aspirin plus …)			0.888
	Clopidogrel (n, %)	1,170 (65.2)	391 (65.4)	
	Ticagrelor (n, %)	479 (26.7)	162 (27.1)	
	Prasugrel (n, %)	146 (8.1)	45 (7.5)	
**Lesion parameters**	First lesion vessel			0.397
	LM (n, %)	435 (24.2)	160 (26.8)	
	LAD (n, %)	876 (48.9)	296 (49.5)	
	Cx/MO (n, %)	320 (17.8)	98 (16.4)	
	RCA (n, %)	133 (7.4)	33 (5.5)	
	RI (n, %)	31 (1.7)	11 (1.8)	
	Lesion localization			0.976
	Ostial (n, %)	64 (3.6)	21 (3.5)	
	Proximal (n, %)	545 (30.4)	187 (31.3)	
	Middle (n, %)	642 (35.7)	209 (34.9)	
	Distal (n, %)	544 (30.3)	181 (30.3)	
	Type C lesion (ref. yes; n, %)	685 (38.2)	212 (35.5)	0.236
	Severe calcification (ref. yes; n, %)	261 (14.5)	88 (14.7)	0.916
	Diffuse disease (ref. yes; n, %)	700 (39.0)	238 (39.8)	0.728
	Kind of bifurcation			0.217
	Distal LM (n, %)	481 (26.8)	174 (29.1)	
	LAD/Diag (n, %)	839 (46.7)	284 (47.5)	
	Cx/MO (n, %)	341 (19.0)	109 (18.2)	
	RCA/PL (n, %)	134 (7.5)	31 (5.2)	

	Medina class			
	1,1,1 (n, %)	605 (33.8)	205 (34.2)	0.128
	1,1,0 (n, %)	587 (32.7)	198 (33.0)	
	1,0,1 (n, %)	174 (9.7)	47 (7.9)	
	0,1,1 (n, %)	87 (4.8)	32 (5.4)	
	1,0,0 (n, %)	158 (8.8)	41 (6.9)	
	0,1,0 (n, %)	92 (5.1)	47 (7.9)	
	0,0,1 (n, %)	92 (5.1)	28 (4.7)	
	Kind of strategy			
	Provisional (n, %)	1,474 (82.1)	490 (81.9)	0.922
	Two stents (n, %)	321 (17.9)	108 (18.1)	
	Use of imaging			
	No (n, %)	1,186 (66.1)	400 (66.9)	0.463
	IVUS (n, %)	586 (32.6)	194 (32.4)	
	OCT (n, %)	23 (1.3)	4 (0.7)	
	Predilatation (ref. yes; n, %)	1,559 (86.9)	531 (88.8)	0.216
	Kind of balloon			
	Conventional (n, %)	1,521 (97.5)	518 (97.5)	0.580
	Angiosculpt (n, %)	18 (1.2)	4 (0.8)	
	Scoring (n, %)	20 (1.5)	9 (1.7)	
	Rotablator (ref. yes; n, %)	106 (5.9)	38 (6.4)	0.689
	Kind of stent on MB			
	Resolute Onyx (n, %)	506 (28.6)	186 (31.3)	0.482
	Xience Alpine (n, %)	454 (25.5)	136 (22.9)	
	Synergy (n, %)	374 (21.0)	131 (22.1)	
	Ultimaster (n, %)	165 (9.3)	50 (8.4)	
	Biomatrix alpha (n, %)	2 (0.1)	2 (0.3)	
	Promus (n, %)	276 (15.5)	89 (15.0)	
	MB lesion diameter (mm)	3.0 [2.8; 3.5]	3.0 [2.8; 3.5]	0.067
	MB lesion length (mm)	22 [16; 28]	23 [16; 28]	0.290
	MB atmospheres (atm)	12 [12; 16]	14 [12; 16]	0.173
	Stent on SB (ref. yes; n, %)	321 (17.9)	108 (18.1)	0.922
	SB lesion diameter (mm)	2.3 [1.0; 2.8]	2.5 [1.0; 2.8]	0.377
	SB lesion length (mm)	28 [20; 33]	28 [20; 33]	0.213
	SB atmospheres (atm)	12 [10; 14]	12 [12; 14]	0.498
	POT (ref. yes; n, %)	1,384 (77.1)	447 (74.7)	0.240
	ATM Post (atm)	20 [16; 22]	20 [16; 20]	0.903
	Final kissing ballon (ref. yes; n, %)	746 (41.6)	248 (41.5)	0.970
**Outcome**	Death (ref. yes; n, %)	103 (5.7)	34 (5.7)	0.962
	Median follow-up at the event (days)	274 [61; 433]	253 [23; 458]	0.852
	Death within 30 days (ref. yes; n, %)	20 (1.1)	9 (1.5)	0.449
	Death within 1 year (ref. yes; n, %)	68 (3.8)	21 (3.5)	0.757
	Death within 2 year (ref. yes; n, %)	94 (5.2)	31 (5.2)	1.000

The table shows patient and lesion parameters in training dataset (n=1,795) compared to internal validation dataset (n=598). Variables are reported as median [interquartile range], or absolute number (percentage, %), as appropriated. Differences were considered significant when $p<0.05$. CKD, Chronic Kidney Disease; PCI, Percutaneous Coronary Intervention; CABG, Coronary Artery Bypass Graft; MI, Myocardial Infarction; EF, Ejection Fraction; STEMI, ST-segment Elevated Myocardial Infarction; NSTEMI, Non ST-segment Elevated Myocardial Infarction; ACS, Acute Coronary Syndrome; DAT, Double Antiaggregant Therapy; LM, Left Main; LAD, Left Anterior Descending; Cx/MO, Circumflex/Marginal; RCA, Right Coronary Artery; RI, Right Intermedius; Diag, Diagonal; PL, Posterior Left; IVUS, IntraVascular UltraSound; OCT, Optical Coherence Tomography; MB, Main Branch; SB, Side Branch; POT, Proximal Optimization Technique.

Table S3. Grid Search: Model and Data Imbalance Correction

<table>
<tr><th>All-cause mortality prediction</th><th>MODEL 1*
Sens Spec, %</th><th>MODEL 2*
Sens Spec, %</th><th>MODEL 3*
Sens Spec, %</th><th>MODEL 4*
Sens Spec, %</th><th>MODEL 5*
Sens Spec, %</th></tr>
<tr><td>SMOTE
(Synthetic Minority Oversampling Technique)</td><td>70.1 / 79.3
[65.7 / 78.8]</td><td>41.6 / 96.8
[29.2 / 96.4]</td><td>73.7 / 78.4
[64.2 / 77.6]</td><td>100.0 /100.0
[1.5 / 98.7]</td><td rowspan="3">24.8 / 87.1</td></tr>
<tr><td>SMOTE
& nearest neighbors</td><td>81.0 / 70.4
[70.8 / 70.0]</td><td>64.2 / 92.2
[43.8 / 91.4]</td><td>87.6 / 67.1
[72.3 / 68.3]</td><td>100.0 / 96.0
[5.8 / 95.6]</td></tr>
<tr><td>Random oversampling</td><td>71.5 / 78.3
[62.8 / 78.0]</td><td>92.0 / 85.4
[60.6 / 84.7]</td><td>72.3 / 77.8
[64.2 / 77.3]</td><td>100.0 / 100.0
[0.0 / 99.9]</td></tr>
</table>

The table shows sensitivity (%) and specificity (%) at training (above) and internal validation (below, square brackets) of 5 different machine learning models and 3 algorithms for dataset imbalance correction in the training cohort (n=1,795). * Model 1 – Linear Discriminant Analysis; Model 2 – Random Forest Regressor; Model 3 –Support Vector Machine (linear kernel); Model 4 – Support Vector Machine (kernel RBF); Model 5 – Isolation forest (algorithms for dataset imbalance correction are not applicable for this classificator). Random forest regressor with random oversampling correction was selected as best model and reported in bold.

Table S4. Tuning of the RAIN-ML model

Tree (n)	Max Splits (n)	Sensitivity (%)	Specificity (%)	Accuracy (%)	MA Accuracy (%)
10	10	67.2	79.7	79.0	73.4
10	20	66.4	81.6	80.7	74.0
10	40	59.1	86.7	85.1	72.9
10	80	51.1	90.9	88.6	71.0
10	160	47.4	92.0	89.5	69.7
10	320	47.4	92.0	89.5	69.7
50	10	67.9	80.3	79.6	74.1
50	20	63.5	83.5	82.3	73.5
50	40	59.1	87.3	85.7	73.2
50	80	51.8	92.5	90.2	72.2
50	160	48.9	93.1	90.6	71.0
50	320	48.9	93.1	90.6	71.0
**100**	**10**	**70.1**	**79.9**	**79.4**	**75.0**
100	20	65.7	83.1	82.1	74.4
100	40	58.4	87.5	85.8	72.9
100	80	46.7	92.3	89.7	69.5
100	160	44.5	93.0	90.2	68.8
100	320	44.5	93.0	90.2	68.8
200	10	69.3	80.4	79.7	74.9
200	20	66.4	83.1	82.2	74.8
200	40	57.7	87.2	85.5	72.4
200	80	46.0	92.5	89.8	69.2
200	160	43.1	93.2	90.3	68.1
200	320	43.1	93.2	90.3	68.1
400	10	67.9	80.5	79.8	74.2
400	20	63.5	83.2	82.0	73.3
400	40	59.1	87.5	85.9	73.3
400	80	45.3	92.6	89.9	68.9
400	160	43.8	93.3	90.4	68.5
400	320	43.8	93.3	90.4	68.5
800	10	68.6	80.7	80.0	74.6
800	20	65.0	83.1	82.1	74.0
800	40	59.1	87.3	85.7	73.2
800	80	45.3	92.5	89.8	68.9
800	160	43.8	93.4	90.6	68.6
800	320	43.8	93.4	90.6	68.6

Hyperparameters (number of classification trees and maximum number of splits) were tuned for the random regressor model to obtain the maximum accuracy in the training cohort (n=1,795). Diagnostic performance (sensitivity, specificity, accuracy and macro-average [MA] accuracy) was assessed by 10-fold cross-validation. A random forest composed by 100 classification trees with a maximum number of splits equal to 10 was selected as best model and shown in bold.

Table S5. Characteristics of the discovery cohort vs. the external validation cohort

	Variable	Discovery cohort (n=2,393)	External validation cohort (n=1,701)	*P*-value
Patient parameters	Age (years)	69 [61; 77]	65 [57; 72]	**<0.001**
	Sex (ref. male; n, %)	1,819 (76.0)	1,329 (78.1)	0.246
	Hypertension (ref. yes; n, %)	1,791 (74.8)	820 (48.2)	**<0.001**
	Hyperlipidemia (ref. yes; n, %)	1,451 (60.6)	N.A.	N.A.
	Diabetes (ref. yes; n, %)	805 (33.6)	319 (18.8)	**<0.001**
	Smoker		N.A.	N.A.
	No (n, %)	1,163 (48.6)		
	Previous (n, %)	729 (30.5)		
	Current (n, %)	501 (20.9)		
	CKD (ref. GFR<60 mL/min; n, %)	538 (22.5)	50 (2.9)	**<0.001**
	Previous PCI (ref. yes; n, %)	792 (33.1)	301 (17.7)	**<0.001**
	Previous CABG (ref. yes; n, %)	126 (5.3)	120 (7.1)	**0.002**
	Previous MI (ref. yes; n, %)	737 (30.8)	339 (19.9)	**<0.001**
	EF at echo (%)	55 [55; 60]	50 [50; 50] *	N.A.
	PCI indication			**<0.001**
	STEMI (n, %)	419 (17.5)	383 (22.5)	
	NSTEMI (n, %)	580 (24.3)	388 (22.8)	
	Unstable angina (n, %)	347 (14.5)	296 (17.4)	
	Stable angina (n, %)	587 (24.5)	634 (37.3)	
	Positive stress test (n, %)	309 (12.9)	0 (0.0)	
	Planned angiography (n, %)	151 (6.3)	0 (0.0)	
	ACS at presentation (ref. yes; n, %)	1,344 (56.2)	1,067 (62.7)	**<0.001**
	STEMI at presentation (ref. yes; n, %)	419 (17.5)	383 (22.5)	**<0.001**
	Kind of DAT (aspirin plus …)			**<0.001**
	Clopidogrel (n, %)	1,561 (65.2)	1,131 (66.4)	
	Ticagrelor (n, %)	641 (26.8)	513 (30.2)	
	Prasugrel (n, %)	191 (8.0)	57 (3.4)	
Lesion parameters	First lesion vessel			**<0.001**
	LM (n, %)	595 (24.9)	179 (10.5)	
	LAD (n, %)	1,172 (48.9)	1,044 (61.5)	
	Cx/MO (n, %)	418 (17.5)	332 (19.5)	
	RCA (n, %)	166 (6.9)	142 (8.3)	
	RI (n, %)	42 (1.8)	4 (0.2)	
	Lesion localization			**<0.001**
	Ostial (n, %)	85 (3.6)	63 (3.7)	
	Proximal (n, %)	732 (30.6)	964 (56.7)	
	Middle (n, %)	851 (35.5)	401 (23.6)	
	Distal (n, %)	725 (30.3)	273 (16.0)	
	Type C lesion (ref. yes; n, %)	897 (37.5)	N.A.	N.A.
	Severe calcification (ref. yes; n, %)	349 (14.6)	397 (23.3)	**<0.001**
	Diffuse disease (ref. yes; n, %)	938 (39.2)	N.A.	N.A.
	Kind of bifurcation			**<0.001**
	Distal LM (n, %)	655 (27.4)	179 (10.5)	
	LAD/Diag (n, %)	1,123 (46.9)	1,045 (61.5)	
	Cx/MO (n, %)	450 (18.8)	334 (19.6)	
	RCA/PL (n, %)	165 (6.9)	143 (8.4)	

	Medina class			
	1,1,1 (n, %)	810 (33.9)	378 (22.2)	
	1,1,0 (n, %)	785 (32.8)	640 (37.6)	
	1,0,1 (n, %)	221 (9.2)	85 (5.0)	
	0,1,1 (n, %)	119 (5.0)	80 (4.7)	**<0.001**
	1,0,0 (n, %)	199 (8.3)	119 (7.0)	
	0,1,0 (n, %)	139 (5.8)	258 (15.2)	
	0,0,1 (n, %)	120 (5.0)	141 (8.3)	
	Kind of strategy			
	Provisional (n, %)	1,964 (82.1)	1,447 (85.1)	**0.004**
	Two stents (n, %)	429 (17.9)	254 (14.9)	
	Use of imaging			
	No (n, %)	1,586 (66.3)	1,648 (96.9)	
	IVUS (n, %)	780 (32.6)	36 (2.1)	**<0.001**
	OCT (n, %)	27 (1.1)	17 (1.0)	
	Predilatation (ref. yes; n, %)	2,090 (87.3)	1,375 (80.8)	**<0.001**
	Kind of balloon			
	Conventional (n, %)	2,039 (97.5)	1,666 (97.9)	
	Angiosculpt (n, %)	22 (1.1)	35 (2.1)	**<0.001**
	Scoring (n, %)	29 (1.4)	0 (0.0)	
	Rotablator (ref. yes; n, %)	144 (6.0)	25 (1.5)	**<0.001**
	Kind of stent on MB			
	Resolute Onyx (n, %)	692 (29.2)		
	Xience Alpine (n, %)	590 (24.8)		
	Synergy (n, %)	505 (21.3)	N.A.	N.A.
	Ultimaster (n, %)	215 (9.1)		
	Biomatrix alpha (n, %)	4 (0.2)		
	Promus (n, %)	365 (15.4)		
	MB lesion diameter (mm)	3.0 [2.8; 3.5]	N.A.	N.A.
	MB lesion length (mm)	22 [16; 28]	15 [10; 22]	**<0.001**
	MB atmospheres (atm)	14 [12; 16]	N.A.	N.A.
	Stent on SB (ref. yes; n, %)	429 (17.9)	442 (26.0)	**<0.001**
	SB lesion diameter (mm)	2.5 [1.0; 2.8]	N.A.	N.A.
	SB lesion length (mm)	28 [20; 33]	N.A.	N.A.
	SB atmospheres (atm)	12 [11; 14]	N.A.	N.A.
	POT (ref. yes; n, %)	1,831 (76.5)	N.A.	N.A.
	ATM Post (atm)	20 [16; 22]	N.A.	N.A.
	Final kissing ballon (ref. yes; n, %)	994 (41.5)	366 (21.5)	**<0.001**
**Outcome**	Death (ref. yes; n, %)	137 (5.7)	39 (2.3)	**0.001**
	Median follow-up at the event (days)	274 [52; 434]	284 [65; 500]	0.714
	Death within 30 days (ref. yes; n, %)	29 (1.2)	6 (0.4)	**0.003**
	Death within 1 year (ref. yes; n, %)	89 (3.7)	22 (1.3)	**<0.001**
	Death within 2 year (ref. yes; n, %)	125 (5.2)	39 (2.3)	**<0.001**

The table shows patient and lesion parameters in the discovery cohort (n=2,393) compared to external validation cohort (n=1,701). Variables are reported as median [interquartile range], or absolute number (percentage, %), as appropriated. Differences were considered significant when $p<0.05$. CKD, Chronic Kidney Disease; PCI, Percutaneous Coronary Intervention; CABG, Coronary Artery Bypass Graft; MI, Myocardial Infarction; EF, Ejection Fraction; STEMI, ST-segment Elevated Myocardial Infarction; NSTEMI, Non ST-segment Elevated Myocardial Infarction; ACS, Acute Coronary Syndrome; DAT, Double Antiaggregant Therapy; LM, Left Main; LAD, Left Anterior Descending; Cx/MO, Circumflex/Marginal; RCA, Right Coronary Artery; RI, Right Intermedius; Diag, Diagonal; PL, Posterior Left; IVUS, IntraVascular UltraSound; OCT, Optical Coherence Tomography; MB, Main Branch; SB, Side Branch; POT, Proximal Optimization Technique.

Table S6. Patient distribution and risk of all-cause mortality

Model Coefficient	Total	No event		Death		All-cause mortality (Risk; %)		
		(N)	(%)	(N)	(%)	30 days	1 year	2 years
0.1	1205	1192	98.9	13	1.1	0.3	0.9	2.3
0.2	1239	1217	98.2	22	1.8	0.7	1.8	3.6
0.3	635	616	97.0	19	3.0	0.5	1.8	3.5
0.4	261	248	95.0	13	5.0	0.8	3.7	8.8
0.5	115	107	93.0	8	7.0	0.9	5.7	13.5
0.6	155	143	92.3	12	7.7	1.3	6.4	14.3
0.7	200	184	92.0	16	8.0	1.0	7.3	16.2
0.8	207	164	79.2	43	20.8	4.4	21.4	53.5
0.9	77	47	61.0	30	39.0	8.1	23.7	72.2
Total	4,094	3,918	N.A.	176	N.A.	N.A.		

The number (N) and the proportion (%) of patients stratified for outcome (No Event *versus* Death) is shown according to the RAIN-ML prediction model in the overall population (n=4,094). Risk of all-cause mortality was stratified according to the RAIN-ML model at 30-days, 1-year, and 2-years follow up. N.A., Not Applicable.

Table S7. Predictive performance and generalizability of the RAIN-ML model

**Predictive Models**	**N**	**Sens**	**Spec**	**PPV**	**NPV**	**Acc**
Training cohort	1,795	82.5	81.0	20.9	98.7	**81.1**
Internal validation cohort	598	67.6	80.5	17.3	97.6	**79.8**
K-center cross validation	2,393	60.6	76.2	13.4	97.9	**75.3**
Risk ranking on the discovery cohort	2,928	71.8	85.9	18.4	98.6	**85.3**
External validation cohort	1,701	56.4	77.6	5.6	98.7	**77.1**
Mixed Discovery & External cohorts	4,094	73.9	79.5	13.9	98.5	**79.3**

Sensitivity (Sens), specificity (Spec), positive/negative predictive value (PPV/NPV), and accuracy (Acc) for all the predictive models (each indicator is derived considering death occurrence as referral outcome). Performance is reported for discovery (training and internal validation), external validation, and mixed cohorts (all patients included in the study; n=4,094). Generalizability of the RAIN-ML model was assessed by K-center cross validation (see methods).

Figure S1. Feature selection

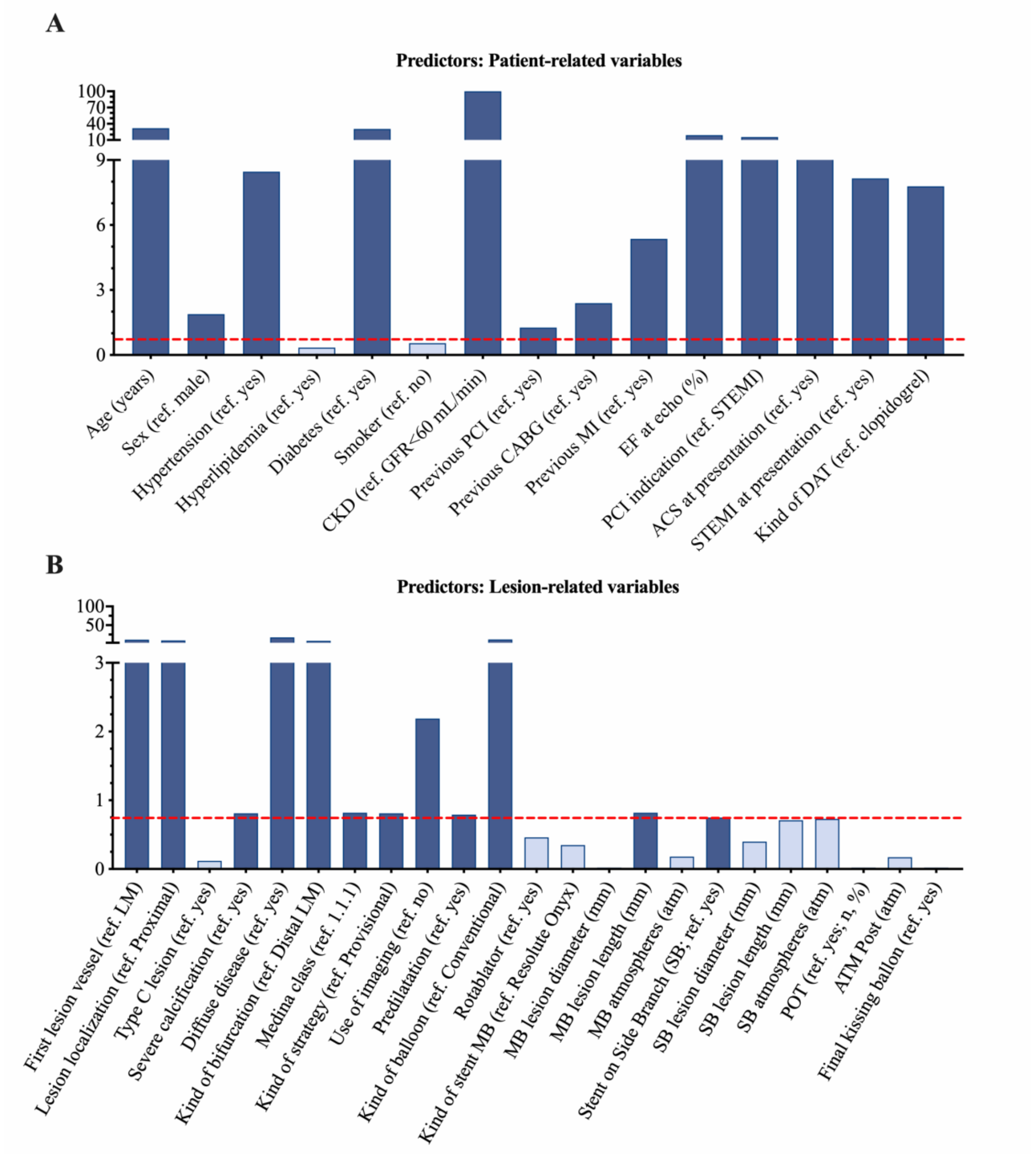

Predictors were selected from patient-related (**A**) and lesion-related (**B**) variables by Fisher Score in the training cohort (n=1,795). Variables with a Fisher coefficient > 0.75 were selected (shown in dark blue) and introduced in diagnostic modeling analysis. CKD, Chronic Kidney Disease; PCI, Percutaneous Coronary Intervention; CABG, Coronary Artery Bypass Graft; MI, Myocardial Infarction; EF, Ejection Fraction; STEMI, ST-segment Elevated Myocardial Infarction; ACS, Acute Coronary Syndrome; DAT, Double Antiaggregant Therapy; LM, Left Main; MB, Main Branch; SB, Side Branch; POT, Proximal Optimization Technique.
